# Endoscopic Coregistered Ultrasound Imaging and Precision Histotripsy: Initial *In Vivo* Evaluation

**DOI:** 10.34133/2022/9794321

**Published:** 2022-07-01

**Authors:** Thomas G. Landry, Jessica Gannon, Eli Vlaisavljevich, Matthew G. Mallay, Jeffrey K. Woodacre, Sidney Croul, James P. Fawcett, Jeremy A. Brown

**Affiliations:** ^1^School of Biomedical Engineering, Dalhousie University, Canada; ^2^Division of Surgery, Nova Scotia Health Authority, Canada; ^3^Department of Biomedical Engineering and Mechanics, Virginia Polytechnic Institute and State University, Virginia, USA; ^4^Department of Pathology & Laboratory Medicine, Dalhousie University, Canada; ^5^Department of Pharmacology, Dalhousie University, Canada; ^6^Department of Surgery, Dalhousie University, Canada

## Abstract

*Objective*. Initial performance evaluation of a system for simultaneous high-resolution ultrasound imaging and focused mechanical submillimeter histotripsy ablation in rat brains. *Impact Statement*. This study used a novel combination of high-resolution imaging and histotripsy in an endoscopic form. This would provide neurosurgeons with unprecedented accuracy in targeting and executing nonthermal ablations in minimally invasive surgeries. *Introduction*. Histotripsy is a safe and effective nonthermal focused ablation technique. However, neurosurgical applications, such as brain tumor ablation, are difficult due to the presence of the skull. Current devices are too large to use in the minimally invasive approaches surgeons prefer. We have developed a combined imaging and histotripsy endoscope to provide neurosurgeons with a new tool for this application. *Methods*. The histotripsy component had a 10 mm diameter, operating at 6.3 MHz. Affixed within a cutout hole in its center was a 30 MHz ultrasound imaging array. This coregistered pair was used to ablate brain tissue of anesthetized rats while imaging. Histological sections were examined, and qualitative descriptions of ablations and basic shape descriptive statistics were generated. *Results*. Complete ablations with submillimeter area were produced in seconds, including with a moving device. Ablation progress could be monitored in real time using power Doppler imaging, and B-mode was effective for monitoring post-ablation bleeding. Collateral damage was minimal, with a 100 *μ*m maximum distance of cellular damage from the ablation margin. *Conclusion*. The results demonstrate a promising hardware suite to enable precision ablations in endoscopic procedures or fundamental preclinical research in histotripsy, neuroscience, and cancer.

## 1. Introduction

Histotripsy is a recent therapeutic ablation approach whereby high negative pressure ultrasonic pulses are focused in tissue, generating small cavitation bubble “clouds.” The rapid expansion and collapse of these bubbles generate high strain capable of homogenizing nearby tissue [1–5]. With the appropriate settings, this occurs without heating and little to no damage outside the main target area. Histotripsy has been shown in preclinical studies to completely destroy tissue in the target area, including in various tumor types [6–8]. Clinical trial results have also been published [9, 10], including a recent liver tumor ablation case study [11].

Neurosurgeons routinely open the skull for procedures but prefer to keep craniotomy size small to minimize trauma and decrease recovery time. This preference makes neuroendoscopy an attractive concept, but surgeons still often choose more open surgical approaches due to the challenges associated with endoscopy [12]. In brain tumor resection surgeries for example, the surgeon advances along a path to the target area using preoperative brain scans (e.g. MRI) as a reference. However, with cranial pressure having been relieved by the craniotomy, the brain can become displaced compared to the preoperative scan—so-called “brain shift”—which can make navigating to the target more difficult and dangerous [13]. Therefore, real-time image guidance, typically by microscope, is essential for accurate navigation. However, conventional optical microscopy only shows features visible at the surface, can be hindered by blood obscuring the surgical field, and has limited usefulness navigating long narrow pathways [14]. Therefore, an endoscopic, depth penetrating, real-time imaging modality would be advantageous.

In response to this, we have developed a 30 MHz high-resolution (40-130 *μ*m) ultrasound imaging system [15–18], with a packaged imaging device diameter of 4 mm. With a long endoscopic form factor, it represents a novel combination of resolution and positioning capability, with imaging penetrating up to 15 mm from the tip. The system also performs power Doppler measurement, providing real-time blood flow information.

In addition to the imaging system, we have also developed the world’s smallest histotripsy transducers [19–23]. The transducers have a 10 mm outer diameter with a 4 mm hole in the center for an imaging endoscope. This is considerably smaller than the next smallest reported histotripsy device at 35 mm diameter with no image guidance [24]. Our devices operate at a higher acoustic frequency (~6 MHz) than previous designs, which should result in a tighter focus and therefore smaller ablation volumes than previously possible. The current focal distance is 6 mm in front of the imaging device, but the focal distance can be adjusted by changing the lens geometry, or even with electronic steering in specially designed devices [25].

For brain tumor resection surgeries, the current gold standard tools used are endoscopic pulsating aspirators (e.g., CUSA or Sonopet), which lack image guidance and produce relatively large contact ablations several mm in diameter [26]. A miniature histotripsy endoscope with a tightly controlled submillimeter ablation zone may provide surgeons with more precision in targeting diseased tissue margins or tissue very close to vital structures that would otherwise be off-limits with the coarse ablation aspirators. There may also be cases where surgeons would prefer to destroy tissue at depth without damaging the intermediate tissue. Such ablations may be possible with a histotripsy endoscope as it is designed to only ablate at the focus of the ultrasound beam. Combining high-resolution ultrasound imaging with simultaneous histotripsy may allow surgeons to precisely target tissues with real-time guidance and immediate treatment monitoring. This may provide an advantage over current standard-of-care aspirators that could improve patient outcomes.

Building upon our earlier precision histotripsy pilot studies [19, 21, 25], we have performed more extensive *in vivo* testing of acute histotripsy in normal rat brains to investigate how various pulse settings, device motion, and target tissues affect the ablation outcome, as well as how ablation progress and postablation changes can be monitored with ultrasound imaging. This study characterizes the device performance and provides a general description of acute *in vivo* brain ablations through ultrasound imaging real-time feedback and histology.

## 2. Results

### 2.1. Benchtop Cavitation and Imaging Performance

**Figure 1:**
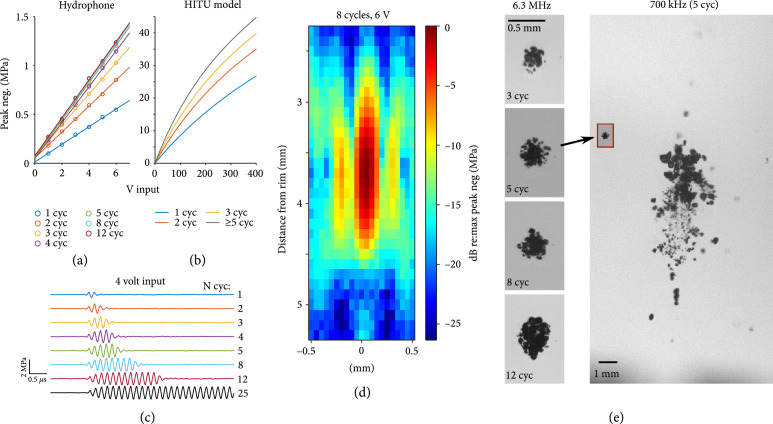
Various benchtop measures of histotripsy device performance. (a–c) Data is from a single device. (a) MPa/V hydrophone measurements for a range of cycles. MPa values are taken as the peak negative pressure value contained in the waveform, with each waveform being an average of 256 trials. (b) Modelled peak negative pressure output at high drive V using the HITU v2.0 script [27], showing the nonlinear output. (c) Waveform data for the 4 V recordings in (a), plus a trace for a 25 cycle pulse, showing the amplitude remains constant well beyond 12 cycles. (d) Spatial profile of peak negative pressure in water for 8 cycles at 6 V in dB relative to the maximum of 1.3 MPa in this case (different device than those in (a–c)). (e) High speed camera images of single pulse bubble clouds in 1% agarose from a 10 mm 6.3 MHz device (but no square hole was in this device) are shown on the left for 3, 5, 8, and 12 cycles at 26 MPa peak negative pressure. For comparison, a 25 MPa 5 cycle pulse bubble cloud is shown for a 700 kHz clinical device (the same used in [9]). Note that the 6.3 MHz and 700 kHz scales are much different to show the details of the 6.3 MHz bubble clouds. The inset red box shows the 6.3 MHz 5 cycle pulse at the same scale as the 700 kHz pulse. All images are captured with 6.74 *μ*s delay from a histotripsy pulse and are from ~20 ms into high pulse repetition frequency (PRF) runs (PRF=1 kHz for 6.3 MHz and PRF=500 Hz for 700 kHz).

Figure 1 shows some of the benchtop measures of histotripsy device performance. Measuring peak negative pressure at different spatial positions with a hydrophone, the −3 dB focal spot lengths and diameters were 0.80 mm and 0.15 mm, respectively, and for −6 dB, the values were 1.20 mm and 0.30 mm. Across three tested devices, the mean peak negative pressure change per V measured at the focal spot for 12 cycle pulses was 0.22 MPa/V (0.20-0.24 range). Low cycle numbers had lower output efficiency slopes, with 1 cycle slope being about 50-70% those of 12 cycles and a steady output being reached by 3-5 cycles. This ramp-up period in multicycle pulse trains is typical and is due to the electromechanical properties of the transducer stack. Using these pressures measured at low V to guide model parameters using HITU simulator v2.0 [27] to account for nonlinear propagation (see *Materials and Methods*), the negative focal pressure was estimated for the different devices and N cycles available. Example model output curves are shown in Figure 1(b). From here onward, estimated MPa values will be given according to the appropriate model curve, but these estimates do not account for attenuation by tissue (~0.3 dB/mm in brain at 6 MHz [28]), or the increase in effective negative pressure due to shock-scatter effects [29].

Optical bubble cloud imaging results showed well-defined dense bubble clouds at various cycle parameters with little to no cavitation observed outside the focus. Prior work has shown that similarly dense and precise clouds result in efficient ablation [30, 31]. For the same pressure, the bubble cloud size appeared to increase between 3 and 5 cycles and then remain stable. Compared to a clinically used 700 kHz bubble cloud, the 6.3 MHz cloud was far smaller and better-defined (Figure 1(e)) which should directly improve treatment precision. Ongoing work is underway to quantitatively characterize the bubble cloud for this device design and investigate bubble behavior more closely.

### 2.2. Brain Imaging and Ablation Dynamics

Basic brain anatomy was clearly visible in the B-mode imaging, especially surfaces, including the layers of the hippocampus. Compared to a previous study using a 40 MHz imaging array [32], the minor loss in resolution with 30 MHz had little impact on the image quality and ability to discern brain anatomy and was well-worth the gain in penetration depth.

During histotripsy and ultrasound imaging, bubble clouds were visible as bright rapidly changing spots. This rapid change is ideal for detection by Doppler processing, as has been shown previously [33]. Therefore, bubble clouds were monitored primarily via power Doppler data, as this signal was usually even stronger and more distinct than in B-mode. Across multiple rats (n=15), cavitation threshold was determined in the cortex. Interpolating threshold values using the HITU v2.0 model output, the mean brain cavitation threshold across all N cycles was −30.7 MPa±2.9 (full range−25.2 to −41.2 MPa), with mean values being consistent across N cycles: within cycle groups from 2-12 cycles, means ranged from −28.8 to −31.9 MPa with no cycle-dependent trend. While these levels are somewhat higher than the brain tissue cavitation thresholds previously observed for lower frequencies [34], the present values may still be overestimated due to tissue attenuation. Furthermore, as frequency increases, cavitation threshold was previously found to increase [35].

**Figure 2:**
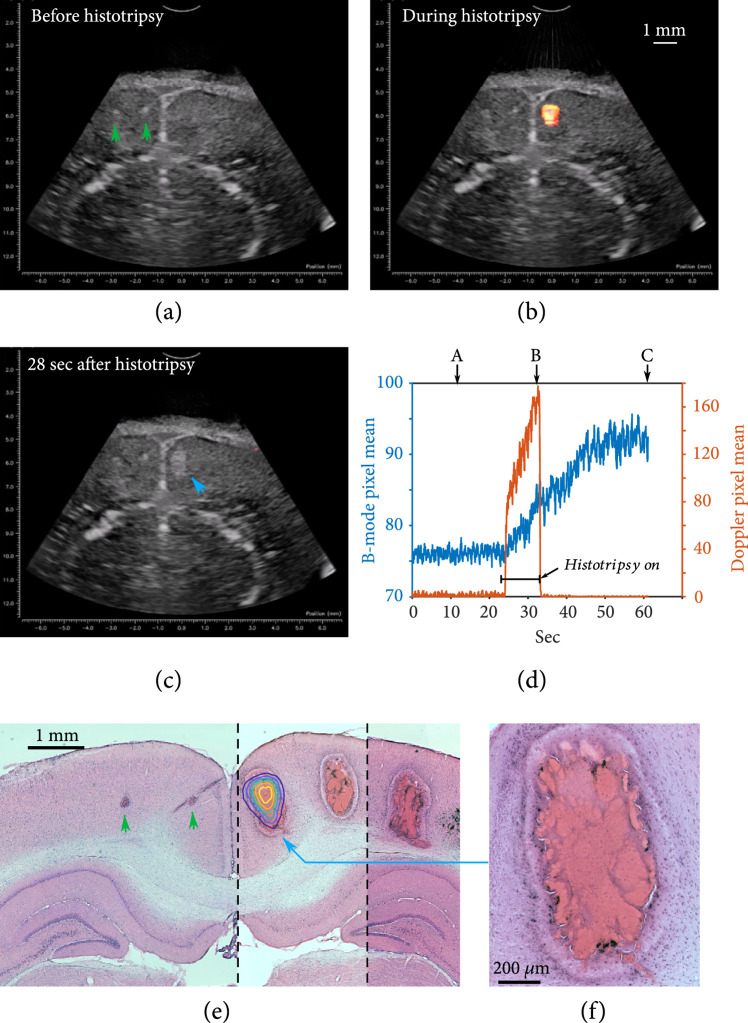
Data showing the real-time imaging changes observed from before (a), during (b), and after histotripsy (c) in normal cortical gray matter. The bubble cloud can be seen in power Doppler mode overlaid on B-mode imaging frames during histotripsy (orange region in (b)). A hyperechoic spot slowly developed in B-mode at the ablation site following histotripsy (arrow in (c)). (d) The plot shows the mean pixel value over time for the B-mode (blue line) and Doppler data (orange line) at the ablation site, with the timepoints for (a–c) indicated. (e) H&E data from the same experiment overlaid with a contour map of the time-averaged Doppler bubble cloud data from the ablation site indicated by the blue arrow in (c) (contour data rotated slightly to match ablation spot). Six histotripsy presentations were done in this experiment from left to right in ~1.8 MPa steps from threshold (T) −1.8 to T+6.6 MPa (T=32.5 MPa in this case; 8 cycles, 10 sec dwell time). Note that “threshold” here refers to the pressure that produced a bubble cloud signal at a different cortical region, not this experimental region, so some variation is expected. The two smaller bright spots in B-mode to the left of midline (green arrows in (a)) are two other very small ablation sites observed in the histology for T+0 and T+1.8 MPa (green arrows in (e)). It was unusual for T+1.8 MPa to produce such a small ablation (first spot left of midline), but a very small spot following T+0 pulsing (left arrow) was seen in about a third of experiments. The presence of these very small spots in B-mode illustrates how well clotted blood is detected with B-mode. (f) The T+3.4 MPa ablation (blue arrow in (e)) at higher magnification. The “halo” region around the ablation described in the text is clearly visible.

After the histotripsy signal was stopped, the bubble cloud disappeared without any sign of residual bubbles. Over the following 10-20 seconds, a hyperechoic area steadily developed at the histotripsy site (Figures 2(c) and 2(d), Movie S1). This effect was consistent across experiments in qualitatively appearing as bright speckle slowly flowing into the area, then becoming fixed in place after 10-20 sec. During the hyperechoic spot development, subtle expansion of the tissue around the site was also often seen, but due to its slow speed and small magnitude, this effect was only clear to the eye during post hoc scrubbing through the video frames at high speed (Movie S1). A future study will quantify the amount of expansion, but a visual estimate of the displacement distance is 100-200 *μ*m. The expansion ceased with the stabilization of brightness and speckle movement. After the initial 10-20 sec postablation, the spots remained stable in B-mode until sacrifice. All of these postablation dynamic changes are likely a result of blood immediately flowing into the space from damaged vessels, then clotting over the 10-20 sec.

**Figure 3:**
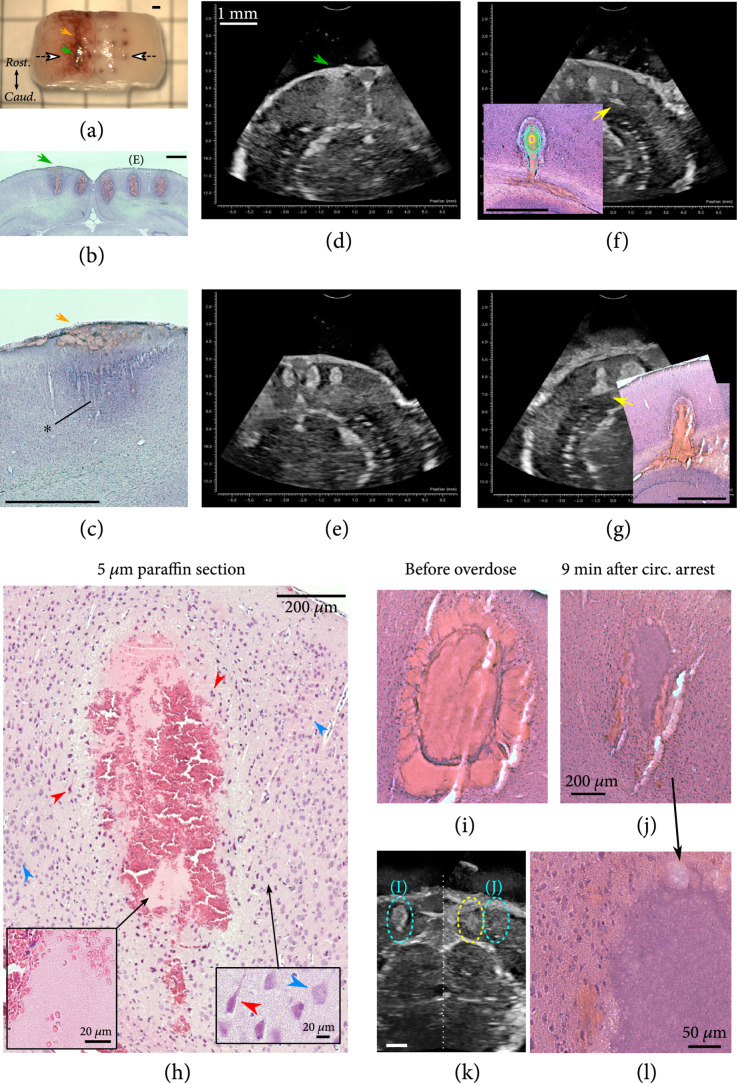
Data showing the agreement between blood seen in histology and B-mode, whether a direct result of histotripsy ((b and e) cortical histotripsy sites), a secondary effect of histotripsy ((f and g) bleeding into white matter tracts), or unrelated to histotripsy at all ((a–d) green and orange arrows). (a) A brain surface injury was accidentally induced during the craniectomy procedure in one animal and was visible as a rostral-caudal line on the surface. The green arrow indicates the surface injury, also highlighted for the same location in histology (b) and B-mode imaging prior to histotripsy (d). The white arrows in (a) indicate the section/imaging plane for (b), (d), and (e). The histotripsy settings for this ablation series were 5 cycles, threshold (T) −1.7 MPa to T+6.2 MPa (T=35.2 MPa) left to right, 3 sec dwell after bubble cloud appearance. (c) At a site with no underlying ablation (orange arrow in (a)), the surface injury is shown in histology without the confounding effect of an ablation below the injury. Darker stain around the injury site was also seen (asterisk). (f) Example showing good agreement between the B-mode patterns that developed after histotripsy with bleeding in the histology ((f) inset). The time-averaged power Doppler contour plot is overlaid on the histology image, showing that the spread of the blood along the white matter tract is a secondary effect. Histotripsy settings were 2 cycles, T+3.5 MPa (35.9 MPa total), 3 sec dwell. (g) Another example of a distinct bright B-mode shape also observed in the histology. This ablation was a 1 mm vertical motion path. Other settings were 2 cycles, T+1.3 MPa (29.9 MPa total), 0.5 mm/s, 3 path passes. (h) Paraffin section showing the extent of injured cells (red arrows) around an ablation. Outside of a 50-100 *μ*m region, neurons appeared healthy (blue arrows). Inside this region, most neurons appeared injured and the extracellular matrix appeared slightly spongy. Histotripsy settings were 3 cycles, T+6.1 MPa (34.5 MPa total), 3 sec dwell. Insets show higher magnification of intact red blood cells (left) and healthy versus injured neurons (right). (i) *In vivo* ablation H&E image showing a marked difference compared to ablation 9 min after the loss of a heartbeat signal following pentobarbital overdose (j). Histotripsy settings were 8 cycles at T+3.7 MPa (33.2 MPa total). (k) The bleeding seen *in vivo* is clear in the B-mode data, whereas there was no change in the 9 min CA condition (compare blue ovals indicating correspondence to (i) and (j)). A less intense signal was seen for a CA ablation 2 min after the heart stopped (yellow oval, histology not shown). (k) Higher magnification image of the CA ablation in (j) showing the uniformity of the homogenate and lack of any cellular structures. All scale bars=1 mm except where indicated.

**Figure 4:**
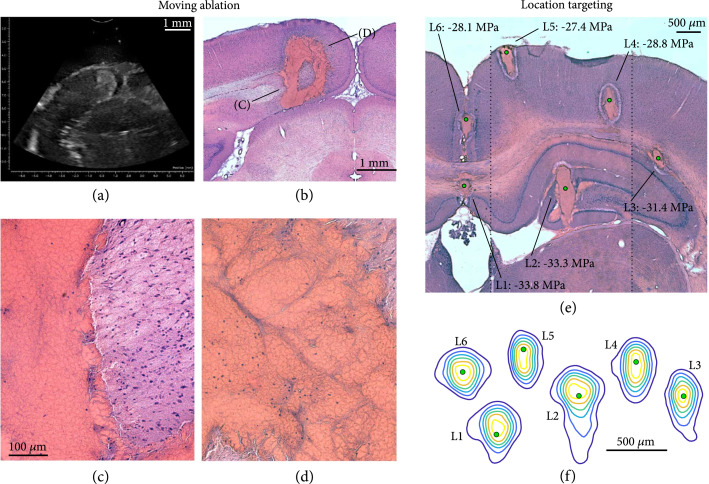
Data demonstrating position and targeting control. (a) Postablation B-mode image from a 1.5 mm tall “D” shaped moving ablation path. The motor-controlled path was a single pass at 0.1 mm/s. Histotripsy settings were 12 cycles at ~38 MPa (~3 MPa above threshold). The hyperechoic post-ablation shape shows good agreement with the histology (b), although the unablated central area was not as clear in B-mode. (c) and (d) show higher magnification images of two locations of the ablation, indicated in (b). The punctate dark purple points in the homogenized area are likely white blood cell nuclei that entered the area post-ablation. (e) Histology showing ablations for six different types of brain locations (L1-L6) in one animal, demonstrating targeting accuracy and the effect local tissue conditions can have on ablations. The small green circles indicate the best estimate of the “target” position on screen before ablation, as determined by the known target coordinate in B-mode relative to nearby anatomical landmarks seen in both B-mode and histology. The pressure used for each site is shown, with these values being in the range 0-0.7 MPa above threshold, determined for each individual site. All ablations were 3 cycles with 3 sec dwell time from when the bubble cloud appeared. (f) Contour plots showing the time averaged power Doppler data intensity for ablations L1-L6 in (e).

The bright postablation shapes corresponded very well to the ablation shapes seen in histology (see Figures 2–4). Bleeding into surrounding structures was sometimes seen in the histology, especially along white matter tracts and fissure surfaces. These were also observed developing in real time in B-mode after histotripsy ceased, with very good agreement between the B-mode bright regions and blood present in the histology (Figure 3). This demonstrates that B-mode was also effective for detecting any bleeding outside of the main ablation site.

A single experiment was performed to determine the acute effects posthistotripsy bleeding had on the ablation site. In this test, histotripsy was performed at one cortical site as usual, followed a minute later by an overdose pentobarbital injection. The breathing/heartbeat signal was monitored, and histotripsy was performed with the same settings at two other sites 2 and 9 min after the heart signal was no longer seen (“circulatory arrest” (CA) condition). The histology for these CA ablations had very different appearances from the *in vivo* site (Figures 3(i)–3(l)). The CA ablation sizes were much smaller in histology (3-6× area reduction) despite no corresponding area difference in the bubble cloud power Doppler signal during ablation. The homogenate did not appear to contain much blood but was stained the same color as the surrounding tissue. The CA ablations were surrounded by small, scattered pockets of blood, likely from blood contained in local vessels that ruptured due to histotripsy. There were similar differences between *in vivo* and CA ablations in the B-mode data in the period following ablation: the *in vivo* postablation period showed the typical emergence of a hyperechoic spot and expansion of the adjacent tissue, whereas no such changes were observed at the 9 min CA site. Interestingly, the 2 min CA site showed some hyperechoic spot development, though not to the same size and brightness as the *in vivo* site (Figure 3(k)), despite there being no major difference in the presence of blood in histology between the two CA sites.

### 2.3. Ablation Appearance in Histology

Histological analysis revealed the ablation was usually an oval or inverted raindrop shape oriented along the beam axis. The central area of the ablation was either a uniformly red color consistent with blood heme pigment or a coarse mixture mixture of two distinctly separate materials—one red and one uniform purple, which was likely the actual brain tissue homogenate. This supposition is supported by results from the CA experiment described above. The 5 *μ*m thin paraffin section results suggest that the red material is largely intact red blood cells (Figure 3(h)). Three other ablation experiments identical to the paraffin-embedded sample but processed as frozen thick sections (35 *μ*m) had the same red/purple swirled appearance seen in other frozen sections. This implies that the material in the paraffin sections is representative of the cellular composition in thick sections, but the individual red blood cells are not distinguishable in the thick sections. In the blood of both the frozen and paraffin sections, white blood cell nuclei were also visible.

Surrounding the central ablation zone was usually a zone of blood nodules over a 10-100 *μ*m width appearing to radiate out from the central zone, giving the ablation margins a bubbled or puckered appearance. Outside this zone was often a 50-100 *μ*m wide halo of darker staining and/or what appeared to be tearing of the brain tissue toward the ablation. This tearing itself was deemed to be a histological artefact due to tissue shrinkage on the slide during alcohol and xylene washes, as this tearing was not at all seen in sections examined prior to staining, nor in the paraffin-embedded brain sample, which was dehydrated and infiltrated with paraffin prior to sectioning. However, there remained in the paraffin sample similar halo regions with distorted and darkly stained cells (Figure 3(h), red arrows) close to the ablation zone, and some possible damage to the extracellular matrix.

The cortical ablation size did not appear to be much influenced by age (young vs. adult) or by pulse parameters, provided the voltage was above cavitation threshold. Based on the qualitative similarity in cortical ablation area across pulse settings above threshold, the area values here are collapsed across ablations for ≥T+20 V (≥T+1-2 MPa) and other pulse settings. The mean area, as defined by the red/purple homogenate region, was $0.33\;\text{m}{\text{m}}^{2}\pm 0.12$. For a fit ellipse to each ablation, the mean major length was 0.94 mm±0.22 and minor width 0.44 mm±0.11. This suggests an average ablated ellipsoid volume of 0.095 mm^3^ (0.041-0.18 mm^3^ if the ellipse dimensions are extended ±1 s.d.). If the periablation region of injured cells is included by adding 100 *μ*m to the ellipsoid radii, the average damage volume becomes 0.24 mm^3^ (0.14-0.40 mm^3^ if the ellipse dimensions are extended ±1 s.d.). Although a thorough quantitative assessment of the agreement between ablation shape in histology and the B-mode hyperechoic postablation spot is beyond the scope of this paper, a comparison between seven typical ablation spots (most ablations visible in Figures 2(e) and 3(b)) found that fit ellipse radius lengths differed by up to 0.27 mm between histology and B-mode. There was a tendency for B-mode spots to be larger (mean difference was +0.12 mm), possibly due to the difference in overall tissue volume from a hydrated and blood-perfused brain *in vivo* versus a dehydrated histological section.

The cortical ablation outcomes for voltages at the threshold value were varied, as expected, given that the value was determined at a different brain site (cortex at Bregma 0.0 to B − 1.0 mm) with slightly different surface geometries present, which could affect the degree of attenuation by the tissue. Out of 23 cortical ablation attempts at the “threshold” level, 7 resulted in no ablation, 8 resulted in a very small (<0.1 mm^2^) ablation spot (e.g., Figure 2(e)), and 8 resulted in a more typical ablation (>0.1 mm^2^).

For ablations with a moving device, no major differences from spot ablations were seen, but a future study will explore this in more detail. Examples shown here of a Dalhousie “D” shape (Figures 4(a)–4(d), Movie S2) and 1 mm vertical line (Figure 3(g)) demonstrate that continuous precision ablation of irregular shapes with a moving device is readily achieved. Ablations performed at sites other than midcortex yielded several interesting findings. When the ablation spot was near a surface or fiber tract, there tended to be postablation bleeding along the surface/tract (e.g., Figure 4(e)), and sometimes bubble cloud presence along these paths as well. Ablations within white matter tracts tended to be more compact. Ablations with a surface interface directly below the target often appeared to be partially “blocked” by the surface, with the ablation and bubble cloud being deflected (e.g., Figure 4(f) L3 contour plot) or appearing denser next to the surface.

### 2.4. Test for Subcavitation Thermal Effects in Tissue

**Figure 5:**
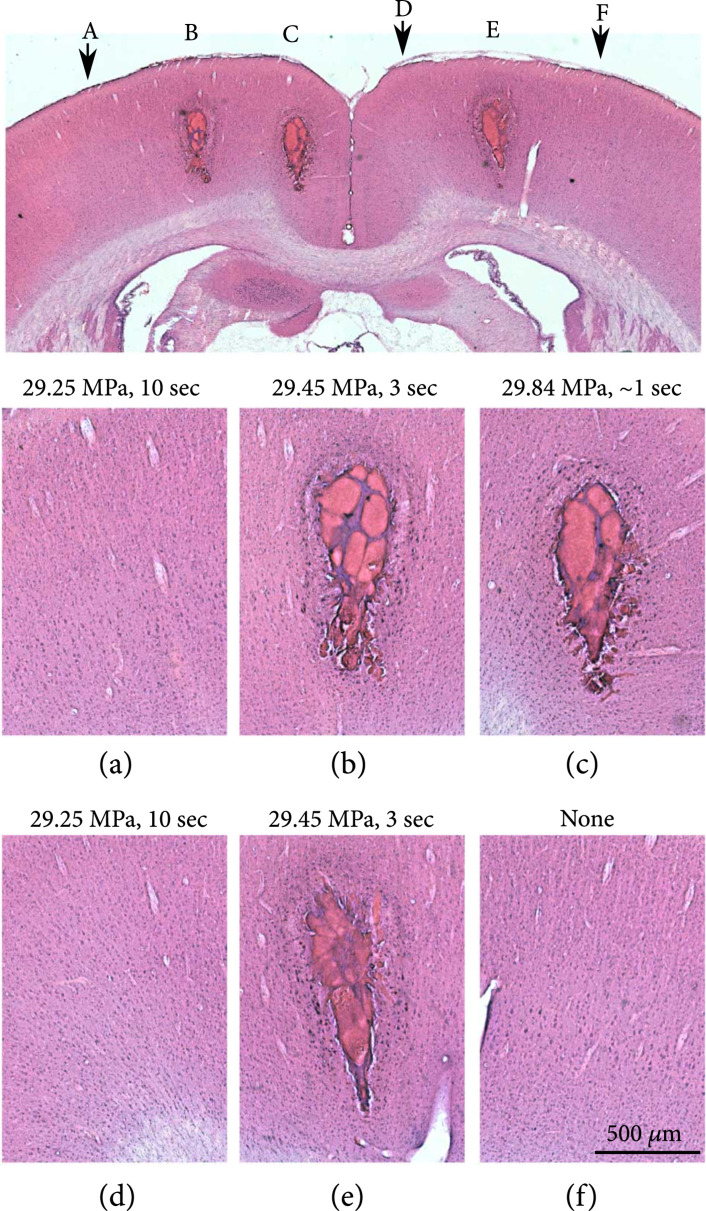
H&E results of a test for thermal effects of the focused acoustic signal. At two cortical sites presented with histotripsy signal (12 cycle pulses, to maximize acoustic energy) just below threshold (T=29.45 MPa) for 10 sec (a, d), the tissue appeared unaffected (compare to that in (f), which did not receive any focused acoustic signal). In contrast, crossing the cavitation threshold resulted in rapid ablation, with a dwell time of ~1 sec (c) resulting in an ablation similar to 3 sec dwell times (b, e).

As our system uses a higher frequency and shorter propagation distance compared to previous studies, the amount of off-target stray cavitation damage was unknown. Previous studies have shown that increasing the pulse repetition frequency (PRF) increases the chance of off-target ablation, but only for lower frequencies (<1 MHz) [30, 35, 36]. In our system, we did not observe any off-target injury, even at the very high 1 kHz PRF. Although the duty cycle is still quite low (0.19% for 12 cycles at 1 kHz), the combination of small focal spot size and higher frequency warranted an investigation into unexpected thermal effects. To investigate this, one experiment was performed wherein two 3 sec ablations were performed at threshold (29.45 MPa, determined at this specific site), one very brief ablation ~1 sec was performed at T+0.4 MPa, and two locations were pulsed at T−0.2 MPa for 10 sec. No bubble clouds appeared at the subthreshold sites and the histology showed no difference compared to control areas that received no focused acoustic dose (Figure 5). There is still potential for subtler thermal effects that do not result in obvious changes detectable in H&E staining, such as heat shock-induced biomolecular responses which deserve a further study, but the present results suggest no major thermal dose damage due to the acoustic signal.

## 3. Discussion

The general concept of this study was to use our system to generate small ablations in normal brain tissue *in vivo* and observe the outcome both in real-time ultrasound imaging and in post hoc histology of the brain tissue. Ablations were performed at high PRF in the cortex of anesthetized adult rats at a range of focal pressures, number of pulse cycles, and total number of pulses. In addition, ablations were performed with both moving and stationary devices, in different target tissue types, and in the cortex of young (27-29 days old) rat brains. The results of this initial study are intended to be a broad overview of outcomes under these different conditions.

The results presented here demonstrate successful submillimeter ablation of *in vivo* brain tissue along with coregistered ultrasound imaging. Furthermore, ablation progress could be accurately tracked using power Doppler data, with B-mode providing excellent postablation feedback regarding the presence of blood at the ablation site and any bleeding into secondary sites. There was no evidence of any major heating effects, and outside of a roughly 100 *μ*m periablation region, brain tissue appeared normal. The repeated success of experiments targeting specific brain locations demonstrates a high degree of positional precision, with likely less than 100 *μ*m error. Quantifying the targeting accuracy with higher precision is difficult because of uncertainties in coregistering histology and B-mode data.

An advantage of using high PRF is that even a brief duration of less than a second is still hundreds of pulses, allowing for ablation with a “real-time” user perception. That is, the user does not need to wait for the execution of a repetitive low PRF pulse routine and can ablate with a more continuous workflow. This is advantageous for a hand-held device. Alternatively, robotically assisted device control may be preferred for executing specific ablation paths or repeatable patterns. Both approaches are compatible with the system described here.

The results of the *in vivo* versus CA experiment were very informative for interpreting the histology. These results suggest that the homogenate observed in CA ablations is representative of the actual homogenized brain tissue, whereas the red material seen at *in vivo* ablations is at least partly blood which migrated into the ablation site. Indeed, closer inspection of *in vivo* ablations usually showed a coarse mix of purple stained material (homogenized brain tissue) and red material (blood). Small nuclei consistent with white blood cells were often observed in the red material, along with intact red blood cells clearly visible in thin sections, whereas no discernable cellular structures could be found in the purple stained material. If both the blood and brain homogenate were fully liquefied for the entire period from ablation to formalin fixation, it would be reasonable to expect a mixture of the two materials to be more homogenous in histology. The fact that there is often a distinct coarsely swirled appearance is best explained by blood flowing into the space, then briefly mixing with the brain homogenate before becoming clotted, locking in the swirled structure. The timeline of posthistotripsy events in the B-mode data—the development of the hyperechoic spot and accompanying tissue expansion, which both stabilize after 10-20 sec—supports this narrative.

An important implication of this conclusion is that the volume of *in vivo* ablated tissue is likely to be smaller than is seen in the histology or B-mode hyperechoic area, as some portion of the “ablation” volume is likely blood and fluids that enter the ablation space from adjacent damaged tissue, expanding the apparent ablation size. Indeed, this is the best explanation for the observation that CA ablations were much smaller than the *in vivo* ablation in the same animal (although this *in vivo* ablation was above average size; Figure 3(i)) despite no corresponding difference in the power Doppler bubble cloud area during ablation. A future study will investigate this effect in more detail by quantitative video analysis of the tissue expansion, which should help quantify the degree of apparent size increase.

The postablation development of a hyperechoic spot, rather than a hypoechoic void, was in contrast with other studies. Our data suggests that any such hyperechoic region corresponded to clotted blood, and its appearance following ablations in this study may be a result of the small ablation size, whereas lower frequency histotripsy generates ablations that are too large to fill via bleeding from local small vessel damage. The fact that these ablations were performed in brain, which is one of the most blood-perfused organs, may also have resulted in more bleeding compared to other organs.

The longer-term reaction to histotripsy in the brain is an important topic for follow-up studies. Based on the results of this study, the ablation site stabilizes fairly quickly—within 20 sec—but it is unclear how the body will respond to this type of brain injury in the long term. Previous studies of histotripsy in pig brains found no adverse reaction to cortical tissue ablation after 72 hours [37] and up to 8 days following intracerebral hemorrhage ablation [38], which were the maximum survival times for those studies. Timepoints even longer than these would be ideal to allow ample time for the stabilization of responses, such as scar formation [39]. Previous small animal studies have shown good tolerance to histotripsy in other organs [6, 40], but the relatively large ablation size produced in previous studies precludes similar studies in the brains of small animals. Large animal (e.g. pig) studies are very resource intensive, limiting the time scale and group size of these studies. Therefore, longer term and higher N brain histotripsy studies would be better performed in small animal models, but this requires smaller ablation volumes to achieve nondebilitating injury extent in the smaller brains. The small and precisely targeted ablations achieved in this study demonstrate that this system could be ideal for preclinical small animal brain ablation studies.

Assessing brain tumor ablation in animal models in acute and longitudinal experiments is another important topic for further study. Brain tumors are not normal brain tissue and have higher stiffness [41–43]. Therefore, there may be differences in the ablation outcomes for tumor tissue if the same histotripsy pulse parameters are used for normal and tumor tissue in the brain. An exciting, related research topic is the immune response to tumor ablation. A number of recent studies have found that histotripsy may induce a much stronger immune response, both local and systemic, than conventional tumor ablation methods [44–48]. Multiple studies outside of the brain have demonstrated posthistotripsy intratumoral T-cell infiltration inducing an immune attack on tumor cells, as well as nontarget tumors in the body—a phenomenon called the abscopal effect [11, 44, 45, 49]. The hypothesized reason for histotripsy excelling in this application is that by mechanically ablating the tissue to a fine slurry, the tumor cells are completely without cellular structure and the antigens and damage-associated molecules are therefore maximally freed for detection by immune cells, but without the biomolecule-degrading effects of thermal ablation, cryoablation, and radio-ablation. However, as with other ablation techniques, histotripsy on its own may not be sufficient to trigger a robust immune system response. Histotripsy paired with an immunomodulatory adjuvant, such as checkpoint inhibitor treatment, seems likely to yield the best outcome [44, 48, 50]. However, as the brain is a unique organ with specialized systems isolating it from the normal blood supply and immune system, it is unclear how the immune response to histotripsy may differ from tumor ablation in other organs.

In conclusion, the system used in this study achieved rapid submillimeter ablation of brain tissue at a focal spot with coregistered real-time imaging feedback, including during continuous device motion. There was no indication of cellular damage beyond a 100 *μ*m periablation region. The ablation edge was well-defined, with irregularities only on the order of tens of micrometers. The ablation shape ranged from elliptical to inverted raindrop shaped, with possible shape distortions near tissue surfaces or when transitioning between white and gray matter. Fabrication and testing of 8 mm diameter devices are currently underway, which would further improve the device utility in neurosurgery. This system could provide surgeons and researchers with a new tool for precisely targeted nonthermal ablative treatments for brain tumors and other neurological disorders.

## 4. Materials and Methods

### 4.1. Experimental and Technical Design

Our ablation and imaging system consisted of four major components: (1) a 10 mm diameter PZT-5A histotripsy transducer with an aluminum lens with a 4×4 mm square hole in the center, (2) voltage drive electronics for histotripsy, (3) a 3.6×3.8 mm 30 MHz imaging array axially aligned and affixed in the square hole of the histotripsy device, and (4) transmit/receive beamforming electronics and display software. The operation of the histotripsy and imaging components was synchronized, so histotripsy could be performed with simultaneous imaging that was relatively artefact-free, allowing monitoring of histotripsy progress and posthistotripsy effects in the tissue.

### 4.2. Device Construction and Coregistration

**Figure 6:**
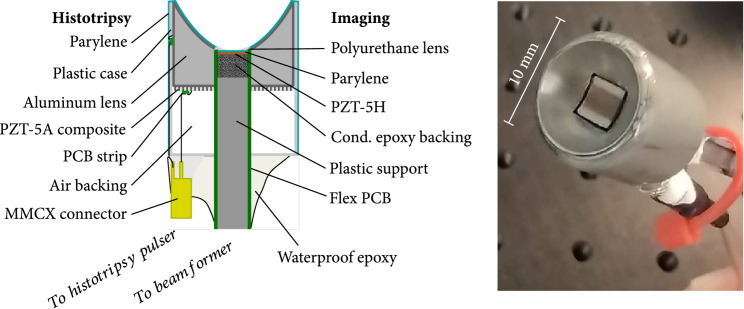
Schematic of the coregistered histotripsy and imaging device, with a front face photograph of a finished prototype device.

The coregistered imaging/histotripsy devices consisted of two independent devices—a 3.6×3.8 mm, 64 element, 30 MHz phased array imaging probe, and 10 mm diameter 6.3 MHz histotripsy device—that were epoxied together to align the focal position of the histotripsy probe in the center of the imaging plane. A schematic and photograph of the finished coregistered device are shown in Figure 6. The only significant imaging device differences from the device detailed previously in Bezanson et al. [16] are that for this study the imaging array was reduced in frequency from 45 MHz to 30 MHz and the element-to-element pitch was increased to 48 *μ*m. These changes were made to gain more penetration depth at the expense of slightly decreased resolution. See *Supplementary Methods: imaging probe construction* for more details.

For the histotripsy device, the fabrication details were essentially the same as those detailed by Woodacre et al. [19]. See *Supplementary Methods: histotripsy probe construction* for more details. Before coregistration with an imaging probe, histotripsy devices were confirmed to be working by presenting to the transducer unipolar negative square wave voltage pulses of 5-10 cycles, 6.0-6.5 MHz, and 1 kHz pulse PRF. The voltage was increased until audible cavitation was observed in deionized water.

For coregistration, the two devices were held by separate XYZ micromanipulators. With the histotripsy device cavitating in water and ultrasound imaging running, the imaging array was lowered into the square hole at the back of the histotripsy device and the depth (Z) position was adjusted until the bubble cloud could be seen at a depth of 6-7 mm in the imaging window. This placed the imaging array face at or just slightly past the bottom of the lens curvature and also placed the histotripsy spot depth approximately in the center of the image window and at the imaging lens focal depth. Epoxy was applied around the back of the histotripsy case and imaging probe shaft to fix the devices together. After curing, the small gap at the lens face/imaging array was sealed with a small amount of silicone (Nusil MED10-6655, Avantor, Radnor, PA).

### 4.3. Histotripsy Device Testing

The histotripsy device was characterized by pressure output measurements using a fiber optic hydrophone (Precision Acoustics, Dorset, UK) during low voltage pulsing in deionized and degassed water to extract a Pa/V conversion factor at the focal spot. This measurement could not be done in water at the high pressures needed for cavitation because the hydrophone would become damaged. However, the low voltage Pa/V measured is useful for assessing the output efficiency of the device and characterizing the acoustic beam. By moving the device with XYZ micromotor positioners, the spatial pressure field was measured. The device output at high voltages up to 400 V in water was simulated using the HITU simulator v2.0 Matlab (Mathworks, Natick, MA) script that was developed at the Food and Drug Administration [27]. The device geometry was modelled as a 10 mm diameter with a 4.4 mm diameter hole to match the area to the 3.9×3.9 mm square hole and 7 mm geometric focal distance. The low-voltage hydrophone data was used to calibrate the total acoustic power parameter for each individual device and number of pulse cycles. The peak negative pressure was extracted, and the simulated −MPa/V curves were used for estimating the negative pressure output at high drive voltages throughout the study.

To capture images of the submillimeter histotripsy bubble cloud, a high-speed camera was used (Photron Nova S12 monochrome, Proprietary Design Advanced CMOS, Photron USA, Inc., San Diego, California, USA) with a 25 mm F2.8 5× Ultra Macro lens at 4.8 : 1 (LAOWA, Hefei, China) attached, giving a resolution of ~5.2 *μ*m per pixel. A continuous light source (GS Vitec Multi-LED QT Light, Multi-LED G8 controller, 320 W power supply, Soden-Salmünster, Germany) was used to backlight images. With the histotripsy device being pulsed at 1 kHz PRF in 1% agarose gel, images were captured at a 6.74 *μ*s delay, as preliminary testing consistently showed fully formed dense clouds at this timepoint under a variety of pulsing conditions. Photron FASTCAM Viewer software (PFV4) was used to record and save bubble cloud frames and videos. Images were analyzed across the range of parameters tested in the *in vivo* studies including 1-12 cycle pulses applied at different peak negative pressures ranging from ~20-26 MPa with a step size of 10 (~1.4 MPa peak negative pressure). Finally, the size and shape of the histotripsy bubble clouds generated from our high precision endoscopic device were compared with an example bubble cloud generated from a 700 kHz clinical histotripsy transducer [9].

### 4.4. Ultrasound Imaging System

The details of the in-house built beamforming hardware and imaging system have been described previously [15]. The imaging software was developed in collaboration with Daxsonics Ultrasound Inc. (Halifax, Canada), featuring analog interface controls, image data processing, power Doppler processing, and the application of some proprietary image enhancement algorithms. The frame rate during experiments was typically 20-25 Hz. The imaging system output a histotripsy trigger pulse approximately every 1 ms, occurring between imaging line transmit events to minimize the presence of electrical artefacts from histotripsy in the imaging data. See *Supplementary Methods: ultrasound imaging system and histotripsy electronics* for more details.

### 4.5. Animals and Study Approval

Twenty-two adult male Wistar rats (282-423 g, mean 354 g; Charles River Laboratories, Wilmington, MA) were used for assessment of acute histotripsy in normal brain tissue. In addition, four young male rats (27-29 days old; 78-97 g, mean 90 g) were also used, as younger nervous system tissue may have different mechanical properties than mature subjects [51], which could potentially affect histotripsy outcomes. All procedures were approved by the Dalhousie University Committee for Laboratory Animal Use (protocols 19-020 and 21-088).

### 4.6. In Vivo Brain Histotripsy

Rats were anesthetized with isoflurane in O_2_ at 1.5 L/min (4% induction, 2-3% maintenance). The head was placed in a stereotaxic frame with ear bars and a snout holder (Kopf Instruments, Tujunga, CA). Breathing rate and heart rate were monitored by the signal from a piezoelectric actuator under the thorax. A circular craniectomy was performed, extending from the skull reference point bregma $\left(B\right)+2.4$ mm to B−10.6, extending laterally to ~2 mm down both sides of the skull, exposing most of the cerebrum. The dura mater was left intact. The craniectomy space and surrounding skull surface were covered in ultrasound gel, as was the lens bowl of a histotripsy/imaging device. Any visible bubbles were removed from the gel. The device was clamped to a micromanipulator and rotated to give a coronal imaging plane. The manipulator was controlled by microstepper motors (Zaber Technologies, Vancouver, Canada) coupled to the manipulator knobs, allowing very fine (10 *μ*m precision) repeatable motion path and speed control.

The rostral-caudal brain position could be readily judged by gross anatomy observed in B-mode imaging alone as compared to a rat brain atlas [52] to within 0.1 mm. After identifying the B0.0 position, other brain target positions were navigated relative to this point. Most experiments used B−2.1,B−3.6,and B−5.1. In this context, an experiment refers to a series of ablations at different positions in the same coronal plane while varying a single parameter, such as voltage. Unless the experimental variable was brain region/tissue type, the ablation was always targeted at the cortex (target depth of 0.8 mm from the surface), as the cortex is the largest and most uniform volume of brain tissue and is at the surface. For experiments that tested histotripsy at different brain regions/tissue types, the target positions were as follows: (1) the corpus callosum at midline (i.e., white matter at the bottom of a sulcus), (2) the hippocampus at CA1 (i.e., gray matter with internal surfaces), (3) deep cerebral white matter (i.e., white matter through normal gray matter), (4) cortical gray matter, (5) cortical surface, and (6) directly in the midline fissure.

In addition, histotripsy initiation threshold measurements were performed in the cortex at a separate region away from the main experimental sites—generally between B0.0 and B−1.0. The threshold value was determined by increasing the voltage in 2 V steps (~0.1-0.2 MPa) and waiting several seconds to allow cavitation to potentially occur, as determined by a bubble cloud appearing in the power Doppler signal. The histotripsy signal intensity used in later cortical ablations in the same animal was often chosen relative to this threshold value and may be given as, for example T−20 V or T+5 MPa if estimated conversion to MPa is possible. Threshold was determined for pulses that were 1, 2, 3, 5, 8, and 12 cycles. However, cavitation was rarely achieved for 1 cycle, up to a limit of 400 V peak-peak, beyond which we risked the damaging the histotripsy device.

On/off histotripsy timing was manually controlled, using a stopwatch to control durations. Ablations were usually a single spot with a stationary position and signal-on dwell duration of either 10 sec of total pulsing time (i.e., 10000 pulses for 1 kHz PRF) or dwell for 3 sec after a bubble cloud first appeared in the Doppler signal. Cavitation typically did not appear until 1-2 sec after signal initiation, so these “3 sec” cases would have usually been ~4000-5000 total pulses delivered to the site. In cases where a bubble cloud did not appear on screen, the histotripsy signal was maintained for 10 sec. Histotripsy during device motion was also tested in several experiments with a variety of motion paths. Most of these paths were executed under motorized control at speeds ranging from 0.1-0.5 mm/s and sometimes multiple passes along the same path. Ultrasound imaging data was saved for at least 10 sec before and at least 30 sec after each ablation without moving the device in order to capture video of the entire histotripsy treatment plus the immediate post-treatment effects in the target region.

After histotripsy experiments were completed, rats were sacrificed with an overdose injection of sodium pentobarbital (150 mg/kg). The brain was then removed and fixed overnight at 4°C in formalin. Due to the time taken for all ablations to be performed in each animal, there was a wide range of time delays between ablation and tissue fixation across ablations, ranging from 5 to 75 min, although the maximum time for most animals was 30 min. After fixation, the brain was sequentially added to 15% sucrose in phosphate buffered saline, then 30% sucrose. The tissue was then cryoembedded in Tissue Plus O.C.T. embedding medium (Fisher Scientific, Waltham, MA) and stored at −80°C. The blocks were sectioned at 35 *μ*m thickness with a cryostat (Leica, Wetzlar, Germany) and stained with hematoxylin and eosin (H&E).

One brain, from a 29-day-old rat, rather than being O.C.T. embedded and cryosectioned at 35 *μ*m, was dehydrated in graded ethanol steps and paraffin embedded, then sectioned on a microtome at 5 *μ*m, and stained with H&E. This was done to help interpret potential differences between the more traditional paraffin embedded H&E sections and thicker cryosectioned H&E samples used in all other brains of this study. H&E sections were examined with a Z1 Axio Imager microscope (Zeiss, Jena, Germany), and for every ablation site, the section that appeared to have the largest area was photographed for further image analysis.

### 4.7. In Vivo Data Analysis

*In vivo* data analysis consisted of ultrasound image/video analysis, histology analysis, and brain cavitation threshold value analysis. In addition to qualitative observations, quantitative analysis included H&E cortical ablation area and cavitation threshold values, as these are basic measures important to demonstrate the small size of ablations and that thresholds generally agree with previous studies. To define the ablation area in histology, the shape was semiautomatically segmented using color thresholding in ImageJ software (NIH), as the distinct red color of the blood heme at the ablation site provided a clear signal of the ablation boundaries. The dimensions of a best fit ellipse to the selection were also recorded.

To examine the bubble cloud power Doppler data beyond single frames, time averages across all ablation frames were generated, background signal from preablation frames subtracted, and intensity contour plots generated. Contour plots were normalized to the maximum Doppler signal intensity present and excluded data<15% of the maximum to exclude noise. These plots could then be overlaid on histology images to examine the level of agreement between Doppler bubble clouds and the resulting ablation. There were sometimes small bumps on the contour plot outer lines on one or both lateral sides of the bubble could signal (e.g., Figure 3(f) inset), but in most cases, these were attributable to a data processing artefact of unknown origin. All figures were generated with Matlab and Inkscape software. Supplementary movies were edited with DaVinci Resolve software (Blackmagic Design, Melbourne, Australia).

## Data Availability

Due to the large size of raw video and image files, all raw data are available by request to the corresponding author.

## Supplementary Materials

Supplementary 1 The Supplementary Movies S1 and S2. Movie S1: B-mode and power Doppler video showing a 3-second stationary cortical ablation. Histotripsy settings were 12 cycles at 33.6 MPa (3.7 MPa above threshold). After histotripsy finishes, the hyperechoic B-mode spot can be seen developing. An inset image of the H&E histology for this spot is shown, illustrating the excellent agreement between the ablation in histology with the hyperechoic B-mode spot. At the end of the data recording, the video frames are scrubbed in forward and reverse several times over the post-ablation period to show the subtle tissue expansion seen in conjunction with the bright spot development. The red arrows indicate two example regions where the expansion can be seen by the bulk movement of the speckle pattern. Throughout the video, a different ablation site can be seen left of midline. This ablation was performed approximately 70 sec before the start of the video, with the same histotripsy settings except pressure was 31.7 MPa. Movie S2: B-mode and power Doppler video showing a 1.5 mm tall “D”-shaped moving ablation path (different experiment from the Figure 4 “D”). The movement speed was 0.1 mm/s. Histotripsy settings were 5 cycles at ~32 MPa (~3 MPa above threshold). Playback speed is 4×. A composite image of the ablation H&E histology is shown at the end.

Supplementary 2 Supplementary Methods: device construction and operating hardware. Additional study Methods are also given in the Supplementary Materials document describing in detail the imaging and histotripsy probe construction steps, as well as the imaging and histotripsy electronics and processing.
