# Supplementary material for: Endoscopic Coregistered Ultrasound Imaging and Precision Histotripsy: Initial In Vivo Evaluation
Source: BME Front. 2022 Jul 1;2022:9794321. doi: 10.34133/2022/9794321 (PMC10521722; doi:10.34133/2022/9794321)
Supplement: Supplementary 2 — Supplementary Methods: device construction and operating hardware. Additional study Methods are also given in the Supplementary Materials document describing in detail the imaging and histotripsy probe construction steps, as well as the imaging and histotripsy electronics and processing. [file 9794321.f2.docx]

**Supplementary Material**

*Supplementary Video Legends*

Movie S1. B-mode and power Doppler video showing a 3 second stationary cortical ablation. Histotripsy settings were 12 cycles at 33.6 MPa (3.7 MPa above threshold). After histotripsy finishes, the hyperechoic B-mode spot can be seen developing. An inset image of the H&E histology for this spot is shown, illustrating the excellent agreement between the ablation in histology with the hyperechoic B-mode spot. At the end of the data recording, the video frames are scrubbed in forward and reverse several times over the post-ablation period to show the subtle tissue expansion seen in conjunction with the bright spot development. The red arrows indicate two example regions where the expansion can be seen by the bulk movement of the speckle pattern. Throughout the video, a different ablation site can be seen left of midline. This ablation was performed approximately 70 sec before the start of the video, with the same histotripsy settings except pressure was 31.7 MPa.

Movie S2. B-mode and power Doppler video showing a 1.5 mm tall “D” shaped moving ablation path (different experiment from the Fig.4 “D”). The movement speed was 0.1 mm/s. Histotripsy settings were 5 cycles at ~32 MPa (~3 MPa above threshold). Playback speed is 4×. A composite image of the ablation H&E histology is shown at the end.

*Supplementary Methods: Imaging probe construction*

The imaging probes were constructed via the following steps:

1) Bulk slabs of PZT-5H (Smart Material, Dresden, Germany) were cut into 25×25 mm squares with a DAD3220 dicing saw (Disco, Tokyo, Japan) and mounted on a glass disc with crystal bond (Electron Microscopy Sciences, Hatfield, PA).

2) The factory electrode was lapped off the piezo using 3 µm aluminum oxide slurry with a lapping machine (Logitech, Glasgow, UK).

3) Using a metal deposition chamber (Mantis Deposition, Oxfordshire, UK), 2 µm of copper was sputtered onto the piezo.

4) The piezo was flipped over and lapped to 52 µm thickness, excluding the 2 µm thick copper electrode on the underside.

5) The lapped surface was electroded as above, but with copper thickness of only 0.8 µm.

6) A backing layer of E-solder conductive epoxy (Von Roll, Augsberg, Germany) was built up to over 2 mm height and cured.

7) The back surface of this layer was then lapped flat to 2 mm total slab thickness.

8) The slab was flipped and the 2 µm copper electrode surface cleaned thoroughly with methanol. The electrode array pattern was then laser etched using a 355nm pulsed picosecond laser (Oxford Laser, Oxfordshire, UK). This pattern consisted of 64 piezo elements 40 µm × 2 mm with 48 µm pitch. Between each element a kerf was laser-diced to separate neighboring elements, and each element was sub-diced using a zig-zag pattern near the centre of the element to suppress lateral modes. Each element ended with a bulbous electrode pad for wire-bonding, with pads alternating between array sides (i.e. 32 pads per side). This array pattern was repeated on the 25×25 mm slab in a grid, usually 4×5 arrays, with at least 0.6 mm separating arrays.

9) This grid of arrays was diced into separate array stacks with a dicing saw.

10) An electrical interconnect mount was prepared using two custom-designed flexible printed circuit boards (PCBs). These 4-layer PCBs each had 32 thick copper traces at the array facing end that led to a 34 element ZIF connector at the cable-facing end (the two additional ZIF elements connected to the ground plane). The 32 thick traces had 96 µm pitch alternating between two internal PCB layers (i.e. 192 µm trace pitch within each trace layer). On the PCB surface near where the array would later be mounted, the ground layer electrode was exposed in a large area. To prepare the PCBs for connection with the array, the tips at the array end were first diced off, exposing the trace cross-sections as ~80×50 µm copper surfaces.

11) The PCBs were bonded to a 3D-printed support stick using epoxy. The PCB tips were positioned just over 2 mm past the end of the stick, leaving a space to insert the array stack with the PCB exposed ground electrode forming the inner surface of the space.

12) Once the PCB epoxy was cured, the inner surfaces of the array space were coated in E-solder conductive epoxy, including the PCB exposed ground layers. An array stack was then inserted into the space and the position adjusted under a microscope to align the array bonding pads with the PCB copper traces. Small dental elastics were used to squeeze the PCBs onto the array during conductive epoxy curing. This step electrically connected the array back face to the PCB ground layer in addition to fixing its position.

13) Using an ultrasonic welding wire-bonder (model 4526, Kulicke & Soffa, Singapore), the PCB traces were connected to the piezo array elements via 15 µm silver wire connections. An impedance analyzer (Agilent model 4294A, Keysight, Santa Rosa, CA) was used to confirm connectivity of each element and broken or poor quality bonds were repeated if possible.

14) The wire bonds were potted with an epoxy resin thickened with aluminum oxide powder.

15) Impedance and phase responses from 10-70 MHz were recorded for several elements across the array using the impedance analyzer. Resonance tended to be in the 35-40 MHz range, with phase peaking from −20° to +5° and impedance at 120-180Ω. Using a KLM model running in Matlab, modelled piezo parameters were adjusted to match the recorded impedance analyzer data. Then the optimal thickness of a parylene-C quarter wavelength matching layer was determined for producing a modelled pulse echo with high amplitude, minimal −6 dB width, and minimal ringing/spurious peaks in the tail region. The optimal parylene thickness was generally in the 20-22 µm range.

16) Parylene-C was deposited to the optimal thickness (±0.1 µm) using a parylene deposition system (Specialty Coating Systems, Indianapolis, IN).

17) An acoustic elevation lens of polyurethane was cast onto the array face using a 7/32” diameter polished Teflon rod.

*Supplementary Methods: Histotripsy probe construction*

The histotripsy probes were constructed via the following steps:

1) Bulk PZT-5A composite samples (5 MHz, 40% volume fraction; Smart Material) were cut into 10.5×10.5 mm squares with a dicing saw.

2) The piezo square was mounted on a glass disc with crystal bond and cut into 10 mm circles using a core drill bit. A ~1 mm hole was also drilled in the center of the circle.

3) A very thin layer of epoxy was spread onto the piezo and the back face of a custom aluminum lens was aligned onto the piezo and cured while under a pressing jig. The lens was electrically continuous with the piezo electrode via direct contact due to the pressing. The aluminum lens was machined using a CNC machine to have a 10 mm diameter, an elliptical curvature focusing to 7 mm from the bottom of the lens bowl (3.7 mm past the lens rim), and a 3.9×3.9 mm square hole in the center (Fig.6).

4) After the piezo and lens were bonded, the small hole in the piezo center was extended to the edges of the square lens hole by hand filing.

5) The piezo/lens was press fit into the front half of a two-part 3D printed plastic casing and the edges around the piezo back face sealed to the case with UV-cured epoxy (68T Optical Adhesive, Norland, Jamesburg, NJ). This case was open at the lens front face and extended back several mm above the piezo back face. There was a 4×3 mm window in the side of the case over the side surface of the lens.

6) Two small squares (~1 mm2) of PCB with exposed surface electrode were cut with a dicing saw, and one square bonded with cyanoacrylate glue to both the piezo back face and the side of the lens at the case window.

7) Several wire bonds were made from one PCB to the piezo back face electrode, and from the second PCB to the aluminum lens side.

8) The wire bonds were potted with aluminum oxide-loaded epoxy, leaving room on the PCB electrodes to solder wires.

9) A thin wire was soldered to each PCB, extending ~15 mm back away from the device face.

10) The back half of the 3D printed case was put into place. This part of the case included a hollow square shaft that aligned with the square lens hole, and a small hole in the base of the case to allow the piezo back face wire through. The lens wire (electrically connected to the piezo front face) ran along the side of the case up to the back of the device, next to the back face wire emerging from the case. All case seams, including inside the hollow square where the plastic case met the piezo back face, were sealed with UV-cured epoxy. The lens plus back face wires were also potted in place with UV epoxy.

11) The two wires were soldered to a female MMCX coaxial connector. The connector was then potted in place. Electrical connectivity to the piezo was confirmed using an impedance analyzer. Due to the lens and thin epoxy layer at the lens/piezo interface, the impedance curve is characteristically spiky, with a maximum resonance in the 6-6.5 MHz range [20].

12) The MMCX connector was covered in putty for protection, and the entire device was coated in 89 µm (roughly a quarter wavelength) of parylene-C.

*Supplementary Methods: Ultrasound imaging system and histotripsy electronics*

The beamformer was designed and developed by our group in collaboration with Daxsonics Ultrasound Inc. (Halifax, NS, Canada) and at a high level it can transmit and receive signals from 64 parallel ultrasound array elements, beamform the data, and transmit the data to a PC via a USB 3.1 port. There is a transmit circuit board that is based on an FPGA (xc7k160ftbg484-2, Xilinx Inc., San Jose, CA) and it controls the transmit pulse frequency, amplitude, and beamforming delays when pulsing the elements. To receive the 64 parallel signals, they are first amplified using low noise time-gain controlled analog circuitry based on eight 8-channel analog boards. The amplified signals are then directed to eight 8-channel data acquisition boards. Each of the eight digitizing boards have an FPGA (xc7k160ftbg484-2) and eight analog-to-digital converters (LTC2262IUJ-12#PBF, Analog Devices Inc., Norwood, MA) that can sample at rates up to 150 MHz. The FPGAs on the 8 digitizing boards upsample, filter, and beamform (delay and sum) the data as it is received and then the beamformed data is transferred to a motherboard in parallel from the 8 digitizing boards. This motherboard also contains an FPGA (xc7k160ftbg484-2) and is synchronized with the transmit circuit board. It is responsible for compounding the data from the digitizing boards and transferring the data to the PC through the USB cable. The software on the PC is based on the Python programming language and generally demodulates the beamformed data, scan converts it, and plots it in real-time. The software was developed by Daxsonics Ultrasound Inc. and also uses proprietary image enhancement processing. Each beamformed frame consists of 128 line angles (±32°) and 4 focal zones over the 12 mm imaging depth.

The histotripsy device drive electronics consisted of a function generator (AFG3101, Tektronix, Beaverton, OR) to control the input pulse waveform, a Z Series 650 V DC high voltage supply (TDK Lambda, Tokyo, Japan) to control the voltage amplitude, and an in-house built board to convert the function generator waveform to unipolar negative voltage pulses at the amplitude set by the DC supply. For all in vivo experiments, the pulse waveform was 6.3 MHz, square-shaped pulses with 40% duty cycle, operating in triggered burst mode with a variable number of cycles. A histotripsy trigger pulse was sent by the beamformer system every 1.06656 ms, starting with that delay after the first transmit pulse for each frame, and every 32 transmits afterward. This corresponds to a first trigger time point between transmit numbers 29/30, followed by another trigger between transmit numbers 61/62, then 93/94, and so on. There are 1024 imaging transmit events and a total of 32 histotripsy trigger pulses sent per imaging frame. Placing the histotripsy trigger pulse between imaging transmit events minimized the presence of the electrical artefact caused by the trigger pulse in the imaging data, although by adjusting the function generator signal output delay, the degree of artefact removal could be customized. It was found to be useful to retain a small amount of artefact (visible as a faint band of lines at near depths; e.g. see Fig.2B, Movies S1 and S2) as this served as a visible indicator of histotripsy on/off time points in post hoc video analysis.

To best visualize the histotripsy bubble cloud during experiments, a rudimentary power Doppler processing method was used. This was done by performing each transmit configuration (i.e. combination of line angle and focal zone) twice per frame, with 1.06656 ms separating each repetition. During the interim, 31 other transmit-receive events occur before repeating the pattern in a 1.06656 ms block, resulting in a constant 1.06656 ms delay between the two measurements for a given transmit configuration. After these two captures are performed for the first 32 transmit configurations, the process is repeated for the next 32 until all 512 configurations (128 lines angles × 4 focal zones) have finished to complete the frame data set. After processing the data for B-mode, the RF amplitude values from the two captures are subtracted and the absolute difference constitutes the power Doppler data. In other words, the processing essentially displays the absolute pixel shade value change over a single 1.06656 ms period per frame for each pixel. To exclude noise, a low value cut-off was adjusted as needed, based on the degree of noise apparent in the display. The power Doppler intensity (pixel value difference) is displayed in color overlaid on the B-mode image, or optionally on its own.
